# Nerol as a Novel Antifungal Agent: In Vitro Inhibitory Effects on *Fusarium oxysporum, Pestalotiopsis neglecta*, and *Valsa mali* and Its Potential Mechanisms against *F. oxysporum*

**DOI:** 10.3390/jof10100699

**Published:** 2024-10-07

**Authors:** Jingyu Ji, Weihu Ma, Jiyuan An, Bowen Zhang, Wenzhuo Sun, Guocai Zhang

**Affiliations:** 1 Heilongjiang Province Key Laboratory of Forest Protection, School of Forest, Northeast Forestry University, Harbin 150040, China; ji@nefu.edu.cn (J.J.); 1098548982@nefu.edu.cn (W.M.); 19845710818@163.com (J.A.); 18348556488@163.com (W.S.); 2 School of Information and Computer Engineering, Northeast Forestry University, Harbin 150040, China; bzhang60@sina.com

**Keywords:** antifungal activity, cell membrane disruption, enzyme inhibition, *Fusarium oxysporum*, nerol

## Abstract

This study explores the in vitro antifungal effects of nerol, a linear acyclic monoterpene alcohol of plant origin, on *Fusarium oxysporum*, *Pestalotiopsis neglecta*, and *Valsa mali*. To further investigate the antifungal mechanism of nerol against *F. oxysporum*, we examined changes in mycelial morphology and cell membrane integrity-related indices, as well as the activities of antioxidant and pathogenicity-related enzymes. The results demonstrated that nerol exhibited significant concentration-dependent inhibition of mycelial growth in all three fungi, with EC_50_ values of 0.46 μL/mL for *F. oxysporum*, 1.81 μL/mL for *P. neglecta*, and 1.26 μL/mL for *V. mali*, with the strongest antifungal activity observed against *F. oxysporum*. Scanning electron microscopy revealed that nerol severely disrupted the mycelial structure of *F. oxysporum*, causing deformation, swelling, and even rupture. Treatment with 0.04 μL/mL nerol led to significant leakage of soluble proteins and intracellular ions in *F. oxysporum*, and the Na^+^/K^+^-ATPase activity was reduced to 28.02% of the control, indicating enhanced membrane permeability. The elevated levels of hydrogen peroxide and malondialdehyde, along with propidium iodide staining of treated microconidia, further confirmed cell membrane disruption caused by nerol. Additionally, after 12 h of exposure to 0.04 μL/mL nerol, the activity of superoxide dismutase in *F. oxysporum* decreased to 55.81% of the control, and the activities of catalase and peroxidase were also significantly inhibited. Nerol markedly reduced the activities of pathogenicity-related enzymes, such as endo-1,4-β-D-glucanase, polygalacturonase, and pectin lyase, affecting fungal growth and virulence. In conclusion, nerol disrupts the cell membrane integrity and permeability of *F. oxysporum*, reduces its virulence, and ultimately inhibits fungal growth, highlighting its potential as an alternative to chemical fungicides for controlling *F. oxysporum*.

## 1. Introduction

*Fusarium oxysporum*, a common phytopathogenic fungus found in soil, is globally prevalent and poses significant risks to a broad spectrum of plant hosts, including tomatoes, bananas, cotton, and chickpeas [1]. This pathogen is capable of causing a variety of plant diseases, such as leaf spot, *Fusarium* wilt, and root rot [2,3]. Notably, *F. oxysporum* can infect not only crops but also trees, with recent reports highlighting its impact on species such as Mongolian pine (*Pinus sylvestris* var. *mongolica*) [4] and its pathogenicity to *Pinus nigra* seedlings in northwest Spain [5]. Furthermore, even in the absence of a host, the microconidia produced by *F. oxysporum* can still persist in the soil for an extended period, complicating efforts to control the fungus [6]. Consequently, the prevalence of diseases inflicted by *F. oxysporum* has led to substantial economic losses in both agricultural and forestry production. These losses extend beyond immediate economic impacts, posing threats to ecological stability and impeding broader social development [7,8,9]. *Pestalotiopsis neglecta* is a significant forest pathogen that can cause black spot blight on *Pinus sylvestris* var. *mongolica* [10] and shoot blight on *Cryptomeria japonica* [11].These diseases severely impact forest health and regeneration. On the other hand, *Valsa mali* is the primary pathogen responsible for the Valsa canker of apple, characterized by rot lesions on branches and trunks, and it is a major cause of economic losses in the apple industry in China [8,12].

The management of *F. oxysporum*, *P. neglecta*, and *V. mali* predominantly depends on synthetic chemical fungicides. However, the extensive use of these synthetic chemical fungicides poses significant environmental and ecological risks. Not only do these fungicides threaten non-target organisms, but they also promote the fungi resistance against fungicides [13,14,15]. Furthermore, the accumulation of these chemicals in animal and human tissues raises significant concerns regarding food safety and public health, leading to increased apprehension about the widespread reliance on chemical pesticides [16,17]. In light of these concerns, there has been a notable shift in recent years towards exploring natural compounds as viable alternatives to synthetic fungicides [18]. A burgeoning body of research highlights the efficacy of plant-derived compounds, which are lauded for their environmental sustainability and safety. Among these, plant extracts and secondary metabolites—like essential oils, alkaloids, terpenoids, and flavonoids—have shown considerable promise in the effective control of plant diseases. These natural compounds represent a compelling direction for future research and development in fungicide alternatives [19,20].

Terpenes, as major constituents of plant volatile oils, have garnered substantial attention in recent years due to their extensive biological activities [21]. Among these, nerol, a linear acyclic monoterpene alcohol, is particularly noteworthy. Nerol, which can be extracted from plants in the Rutaceae family, is a predominant component of various commercially available essential oils, such as those derived from orange leaves, lemons, and grapefruits [22,23]. Recognized for its safety profile and distinctive rose fragrance, nerol has been approved by both the US Food and Drug Administration (FDA) and the Joint Expert Committee on Food Additives (JECFA) as a safe food-grade flavoring agent [22,24]. This recognition has facilitated its widespread application across multiple industries, including medicine [25], cosmetics, and food [24]. Recent research highlights nerol’s considerable potential in antimicrobial applications, particularly in inhibiting bacterial and fungal growth. For instance, previous studies have shown that nerol markedly inhibits the growth of *Aspergillus niger*, curbing both mycelial expansion and conidial germination [26]. Similarly, another study revealed nerol was effective against *Candida albicans* by altering cell membrane integrity [27]. Nerol has been shown to impede the growth and development of *Ceratocystis fimbriata* by damaging mitochondrial membranes and inducing reactive oxygen species (ROS) accumulation within cells [28]. Furthermore, nerol exhibits notable antibacterial activity against plant pathogenic bacteria such as *Agrobacterium tumefaciens* and *Clavibacter michiganense* [29]. These studies underscore the potential of nerol as a potent bactericide and fungicide.

Despite these promising findings, nerol has not yet been registered for use against fungal pathogens associated with forest diseases, and its antifungal properties and mechanisms of action against *F. oxysporum* are not yet fully understood. To address these gaps, this study compares the inhibitory effects of nerol on the mycelial growth of *F. oxysporum*, *P. neglecta*, and *V. mali*, revealing that *F. oxysporum* was more sensitive to nerol. Furthermore, we investigated the potential antifungal mechanisms of nerol against *F. oxysporum*. The antifungal mechanism was evaluated through the following indicators: (1) changes in mycelial biomass and microconidia germination rate; (2) assessment of cell membrane integrity of microconidia and mycelial surface morphology using fluorescence microscopy and scanning electron microscopy; (3) changes in relative conductivity and extracellular soluble protein content; (4) alterations in Na^+^/K^+^-ATPase activity; (5) changes in hydrogen peroxide (H_2_O_2_) and malondialdehyde (MDA) levels; (6) activities of superoxide dismutase (SOD), peroxidase (POD), and catalase (CAT); and (7) activities of endo-1,4-β-D-glucanase (EG), polygalacturonase (PG), and pectin lyase (PL).

The aim of this study is to provide a theoretical foundation for the use of nerol as a novel natural antifungal agent, particularly for the control of *F. oxysporum*.

## 2. Materials and Methods

### 2.1. Chemicals and Microbiological Media

Chemicals: Nerol (98%) was procured from Shanghai Yien Chemical Technology Co., Ltd. (Shanghai, China). The assay kits for superoxide dismutase (SOD), peroxidase (POD), catalase (CAT), endo-1,4-β-D-glucanase (EG), malondialdehyde (MDA), polygalacturonase (PG), hydrogen peroxide (H_2_O_2_), pectin lyase (PL), and Na^+^/K^+^-ATPase were sourced from Grace Biotechnology Technology Co., Ltd. (Suzhou, China). All other chemicals utilized in this study are of analytical grade and purchased from Yuanye Biotechnology Co., Ltd. (Shanghai, China).

Microbiological Media: Potato Dextrose Agar (PDA) and Potato Dextrose Broth (PDB) prepared with fresh potatoes purchased from local supermarkets (Harbin, China).

### 2.2. Pathogens

The *F. oxysporum*, *P. neglecta*, and *V. mali* strains were purchased from the China Forestry Culture Collection Center (CFCC) and obtained from the Northeast Forestry University in Harbin, China. These fungi were cultivated on PDA at 25 °C [4,30,31]. To prepare the microconidia suspension of *F. oxysporum*, sterile water was dispensed using a plastic pipette and applied to the surface of a five-day-old *F. oxysporum* colony. The plate was rinsed gently, and the resulting suspension containing microconidia and mycelia was collected. The mycelia were then filtered out using absorbent cotton, and the microconidia suspension was obtained. The microconidia density in the suspension was diluted to 1 × 10⁶ CFU/mL for subsequent tests.

### 2.3. Effects of Nerol on Mycelial Growth

The inhibition action of nerol on *F. oxysporum*, *P. neglecta*, and *V. mali* mycelial growth was evaluated using the method of quantifying mycelial radial growth [32]. Nerol was first diluted with 1% Tween-80 and then sterilized through a 0.22 µm MCE syringe filter. Under aseptic conditions, sterilized nerol solution was added to molten PDA and mixed to prepare media with final concentrations of 4, 2, 1, 0.5, and 0.25 µL/mL. Fungal mycelial plugs (diameter, 5.0 mm) obtained from a five-day-old fungal colony were inoculated into the center of Petri dishes (diameter, 60 mm) containing PDA. Sterile water served as the control, 1% Tween-80 was used as the solvent control, and bromothalonil (4 µg/mL) was used as the positive control. The Petri dishes were sealed with parafilm (PM996, Bemis, Beijing, China) and subsequently positioned in a constant temperature incubator (25 °C). After 7 d of culture, colony diameter was documented. The EC_50_ of nerol against *F. oxysporum*, *P. neglecta*, and *V. mali* was determined using probit analysis in SPSS 26.0 software (IBM Corp, Armonk, NY, USA). Based on the results, the most sensitive fungal species among the three was selected for further tests of its antifungal mechanisms. Each experimental treatment was replicated thrice. The rate of inhibition was determined by applying the following formula:

$$\mathrm{Inhibition}\;\mathrm{rate}\left(\%\right)=\frac{\left(\mathrm{Colony}\;\mathrm{diameter}\;\mathrm{in}\;\mathrm{control}-\;\mathrm{Colony}\;\mathrm{diameter}\;\mathrm{in}\;\mathrm{treatment}\right)}{\mathrm{Colony}\;\mathrm{diameter}\;\mathrm{in}\;\mathrm{control}}\;\times \;100$$

### 2.4. F. oxysporum and Nerol Interaction

#### 2.4.1. Mycelial Biomass

Biomass was quantified using the mycelial dry weight method. Nerol was evaluated at concentrations of 0.0025, 0.005, 0.02, 0.04, and 0.08 μL/mL, with sterile water serving as the control and 1% Tween-80 as the solvent control. Five mycelial plugs (diameter 5.0 mm) were extracted from a five-day-old fungal colony and inoculated into separate culture bottles containing 80 mL of PDB. The cultures were initially incubated at room temperature for 12 h. Subsequently, they were transferred to a shaking incubator and agitated at 150 rpm for 72 h (25 °C). After the incubation period, the mycelia were collected by filtration through a sterile silk cloth, placed in an electric-heated blast drying oven at 45 °C, and dried for 24 h to a constant weight. The dried mycelia were then weighed to determine the biomass. Each experimental treatment was replicated three times to ensure reproducibility.

#### 2.4.2. Microconidia Germination

The effect of nerol on the microconidia germinability of *F. oxysporum* was evaluated using nerol concentrations of 0.5, 1, 2, 4, and 8 μL/mL. An equivalent volume of sterile water was used instead of nerol as the control, with a 1% Tween-80 solution serving as the solvent control. Specifically, the microconidia suspension containing PDB was mixed with nerol and then dropped onto a hemocytometer (XB-K-25), which was then incubated at 25 °C for 12 h in a controlled temperature incubator. After incubation, five circular fields of view were randomly observed under the microscope. Approximately 200 microconidia were randomly chosen in each field of view. The number of germinating microconidia was then counted and documented if the length of the germ tube was equal to or exceeded the length of the microconidia [26]. The microconidia germination inhibition rate was determined by applying the following formula:

$$\mathrm{Inhibition}\;\mathrm{rate}\;\left(\%\right)=\frac{(\mathrm{Total}\;\mathrm{number}\;\mathrm{of}\;\mathrm{microconidia}-\mathrm{Number}\;\mathrm{of}\;\mathrm{microconidia}\;\mathrm{germinated})}{Total\;number\;of\;microconidia}\;\times \;100$$

#### 2.4.3. Scanning Electron Microscopy Observations

To assess the impact of nerol on the mycelial surface morphology and structure of *F. oxysporum*, scanning electron microscopy (SEM) was utilized. The nerol concentration utilized for this study was 0.46 μL/mL (EC_50_). *F. oxysporum* mycelial plugs (diameter 7.5 mm) were taken from a five-day-old colony and inoculated into the center of Petri dishes (diameter 90 mm) containing PDA supplemented with 0.46 μL/mL nerol. In the control plates, an equivalent volume of 1% Tween-80 solution was used to replace nerol. A coverslip was inserted into the PDA medium at a 45-degree angle and incubated at 25 °C for approximately 7 d. After incubation, mycelia adhering to the coverslip were collected. The collected mycelia were then processed according to the protocol detailed by Ji et al. [33]. Specifically, mycelia were fixed with 4% glutaraldehyde for 12 h at room temperature, followed by dehydration using a graded series of ethanol, with each concentration applied for 15 min. The samples were then vacuum freeze-dried for 12 h and then coated with gold using a sputter coater (EMITECH K575X, Kent, UK). Samples were kept in a desiccator until examination with an Apreo S Scanning Electron Microscope (Thermo Fisher Scientific, Waltham, MA, USA) operated at 2000× and 5000× magnifications.

#### 2.4.4. Membrane Integrity

##### Staining of Microconidia with Propidium Iodide (PI)

A PI staining test was performed to assess membrane integrity in *F. oxysporum* microconidia exposed to nerol. The microconidia suspension was treated with 0.46 μL/mL nerol at 25 °C for 8 h, with an equal volume of 1% Tween-80 solution used as the control. The PI solution was diluted to 50 μg/mL in PBS buffer (0.1M, pH 7.4) and mixed with the treated microconidia suspension at a 1:1 ratio. The mixture was incubated at 25 °C with shaking at 150 rpm for 6 h. After incubation, the suspension was centrifuged (5000× *g*, 4 °C), and the pellet was collected, washed with PBS buffer, and resuspended in it. A drop of this suspension was placed onto a coverslip for observation and imaging under a fluorescence microscope (Axio Observer 3, Jena, Germany). The percentage of stained microconidia was then quantified. Each experimental treatment was replicated three times to ensure reproducibility. The microconidia staining rate was determined by applying the following formula:

$$\mathrm{Microconidia}\;\mathrm{staining}\;\mathrm{rate}\;(\%)\;=\frac{\mathrm{Number}\;\mathrm{of}\;\mathrm{stained}\;\mathrm{microconidia}}{Total\;number\;of\;microconidia}\;\times \;100$$

##### Measurement of Extracellular Relative Conductivity, Release of Soluble Proteins, and Na^+^/K^+^-ATPase Activity

Mycelial plugs (diameter 5.0 mm) obtained from a five-day-old colony were inoculated into PDB in conical flasks. Nerol was added to the flasks at final concentrations of 0.01, 0.02, and 0.04 μL/mL, with an equal volume of 1% Tween-80 solution as the control. After standing at 25 °C for 12 h, the conical flasks were transferred to a shaker incubator at 25 °C and 150 rpm for 72 h. Subsequently, the culture was filtered using sterile silk cloth to collect the mycelia and supernatant. The mycelia were washed three times with 0.9% NaCl solution, dried under sterile conditions, and then frozen for Na⁺/K⁺-ATPase activity assays. The supernatant was used to measure extracellular relative conductivity and soluble protein content. Extracellular relative conductivity was determined using a conductivity meter (DDS-307, Laiqi Instrument Factory, Shanghai, China). Na⁺/K⁺-ATPase enzyme activity was measured according to the instructions of the Na⁺/K⁺-ATPase activity assay kit and utilizing a microplate reader (SuPerMax 3100, Shanghai, China). Soluble protein content was quantified using the Bradford method [34]. Each experimental treatment was replicated three times to ensure reproducibility.

##### Determination of H_2_O_2_ and MDA Concentration

Mycelial plugs (diameter, 5.0 mm) obtained from five-day-old cultures were inoculated into conical flasks containing PDB. Nerol was added to the flasks to achieve a final concentration of 0.04 μL/mL, with an equal volume of 1% Tween-80 solution serving as the control. After incubating the flasks at 25 °C for 12 h, they were transferred to a shaking incubator at 25 °C and 150 rpm for 72 h. At designated time points (12, 24, 36, 48, and 60 h) after the 72 h incubation, mycelia were collected and filtered using sterile silk cloth to remove the PDB. The mycelia were then washed three times with 0.9% NaCl solution, dried under sterile conditions, and frozen for further analysis. According to the protocols provided by the commercial assay kits, the concentrations of MDA and H_2_O_2_ were quantified using a microplate reader (SuPerMax 3100, Shanghai, China). Each experimental treatment was performed in triplicate to ensure reproducibility.

#### 2.4.5. Evaluation of Nerol’s Effect on Activity of *F. oxysporum* Enzyme

Mycelial plugs (diameter 5.0 mm) obtained from a five-day-old colony were inoculated into conical flasks containing PDB. Nerol was added to achieve a final concentration of 0.04 μL/mL, with an equal volume of 1% Tween-80 solution as the control. After standing at 25 °C for 12 h, the flasks were transferred to a shaker at 25 °C and 150 rpm for 72 h. At specific time points (12, 24, 36, 48, and 60 h), mycelia were collected and filtered through sterile silk cloth, and both the mycelia and supernatant were collected. The mycelia were washed three times with 0.9% NaCl solution, dried under sterile conditions, and then frozen for the measurement of POD, SOD, and CAT activities, while the supernatant was used to determine EG, PG, and PL activities. Enzyme activities were measured according to the instructions provided with the respective enzyme activity assay kits using a microplate reader (SuPerMax 3100, Shanghai, China). Calculations and expressions of enzyme activities followed the guidelines of each kit. To ensure data reliability, all treatments were replicated three times.

### 2.5. Data Analysis

The experimental data underwent statistical analysis to derive meaningful interpretations. The mean values and standard deviations (n = 3) were computed with Excel 2021 software (Microsoft Company, Redmond, WA, USA). ANOVA was conducted utilizing SPSS 26.0 software (IBM Corp, Cambridge, MA, USA), with significant mean differences determined by the Tukey test. Graphs and visual representations of the data were created using GraphPad Prism 9.4.1 (GraphPad Software Company, San Diego, CA, USA).

## 3. Results

### 3.1. Effect of Nerol on Mycelial Growth of F. oxysporum, P. neglecta, and V. mali

**Figure 1:**
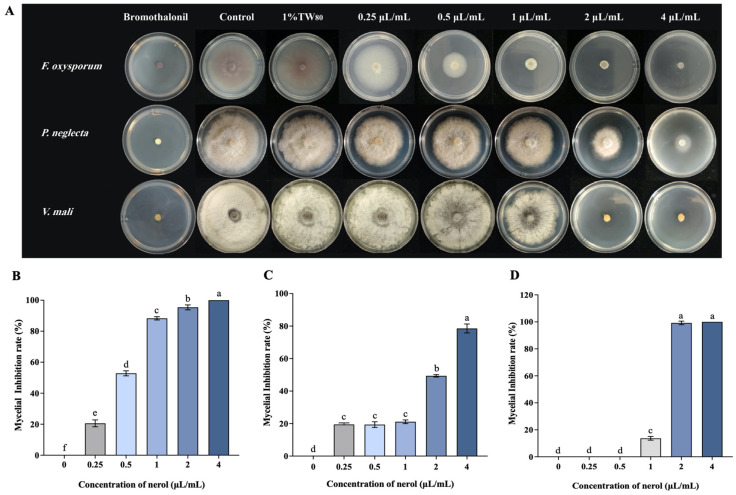
Interaction among the selected target fungi and different nerol concentrations: colony morphology (**A**) and the mycelial inhibition rate of *F. oxysporum* (**B**), *P. neglecta* (**C**), and *V. mali* (**D**). Histograms represent the mean (n = 3) ± standard error. Values accompanied by the different letters are significantly different (*p* ≤ 0.05) according to the Tukey test.

Nerol demonstrated varying levels of suppressive effects on the mycelial growth of *F. oxysporum*, *P. neglecta*, and *V. mali* (Figure 1). At 4 μL/mL concentration, nerol entirely inhibited the mycelial growth of *F. oxysporum* and *V. mali*, achieving a 100% inhibition rate (Figure 1B,D). Specifically, at 0.25, 0.5, 1, 2, and 4 μL/mL concentrations, nerol significantly suppressed the mycelial growth of *F. oxysporum* in a concentration-dependent manner (Figure 1B). For *P. neglecta*, significant inhibition was observed at concentrations of 0.25, 0.5, 1, 2, and 4 μL/mL, although there was no significant difference between the 0.25, 0.5, and 1 μL/mL treatment groups (Figure 1C). In contrast, for *V. mali*, no inhibitory effect was observed in the 0.25 and 0.5 μL/mL treatment groups, while nerol concentrations of 1, 2, and 4 μL/mL significantly inhibited mycelial growth (Figure 1D). The EC_50_ of nerol for *F. oxysporum* was determined to be 0.46 μL/mL, whereas, for *P. neglecta* and *V. mali*, the EC_50_ values were 1.81 μL/mL and 1.26 μL/mL, respectively.

### 3.2. F. oxysporum and Nerol Interaction

#### 3.2.1. Mycelial Biomass and Microconidia Germination

**Figure 2:**
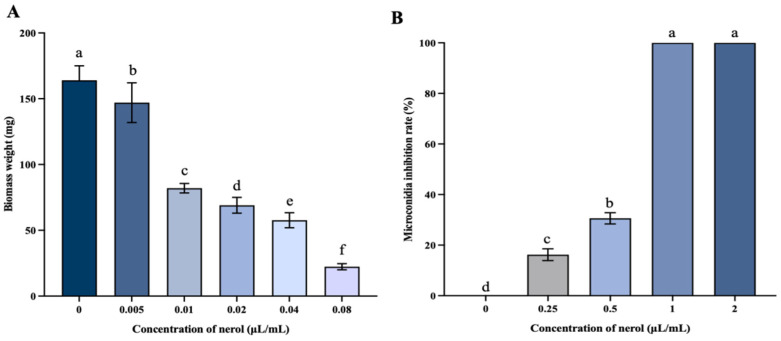
Effects of nerol on the biomass (**A**) and microconidia germination (**B**) of *F. oxysporum*. The bars represent the standard error of the mean (n = 3). Histograms represent the mean (n = 3) ± standard error. Values accompanied by the different letters are significantly different (*p* ≤ 0.05) according to the Tukey test.

As depicted in Figure 2A, mycelial biomass exhibited a significant decline as nerol concentrations increased compared to the control (*p* < 0.05). The control group displayed a dry weight of the mycelium of 172 mg, whereas, in the treatment group with 0.08 μL/mL nerol, the dry weight drastically reduced to 22.33 mg, representing only 12.98% of the dry weight of the control.

In Figure 2B, nerol significantly suppressed the microconidia germination of *F. oxysporum* (*p* < 0.05). At concentrations of nerol below 1 μL/mL, the inhibition rate of microconidia germination increased with higher concentrations of nerol. The inhibition rate achieved by 0.25 μL/mL nerol was 16.24%. Upon reaching a concentration of 1 μL/mL, the inhibition rate peaked at 100%. These data underscore the strong inhibitory impact of nerol on the microconidia germination of *F. oxysporum*.

#### 3.2.2. Nerol Induces Morphological Changes and Abnormal Growth in *F. oxysporum* Mycelia

**Figure 3:**
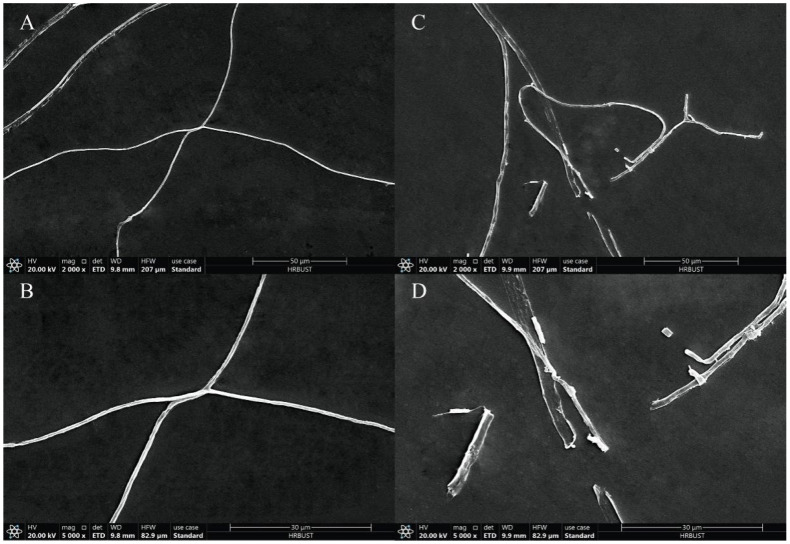
Morphology of *F. oxysporum* hyphae treated with 1% Tween-80 (**A**,**B**) or nerol at its EC_50_ concentration (**C**,**D**) observed through scanning electron microscopy (SEM) at 2000× (**A**,**C**) and 5000× (**B**,**D**) magnifications.

As shown in Figure 3A,B, the untreated mycelium exhibited a smooth surface and a complete structure, presenting a uniform and full linear shape. In contrast, the mycelium treated with 0.46 μL/mL nerol lost its smoothness; the surface became rough, and the mycelium collapsed, became flat, exhibited abnormal bulges, and even showed severe fractures (Figure 3C,D).

#### 3.2.3. Nerol Affected the Permeability of Membrane in *F. oxysporum*

**Figure 4:**
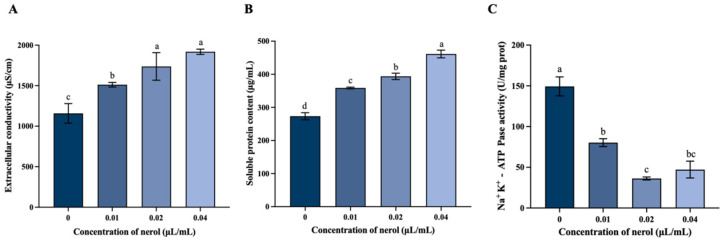
Effect of different concentrations of nerol treatment on relative conductivity (**A**), soluble protein content (**B**), and Na⁺/K⁺-ATPase activity (**C**). Histograms represent the mean (n = 3) ± standard error. Values accompanied by the different letters are significantly different (*p* ≤ 0.05) according to the Tukey test.

The relative conductivity of *F. oxysporum* exposed to nerol (0.01, 0.02, and 0.04 μL/mL) was significantly elevated relative to the control (Figure 4A, *p* < 0.05). Specifically, the conductivity in the 0.04 μL/mL nerol treatment group reached 1918.50 μS/cm, representing a 1.72-fold increase compared to the control group.

According to Figure 4B, the levels of soluble proteins in the 0.01, 0.02, and 0.04 μL/mL nerol treatment groups were markedly elevated compared to the control group (*p* < 0.05). At 0.04 μL/mL nerol, the extracellular soluble protein content increased to 461.43 μg/mL, representing a 1.70-fold rise relative to the control.

Figure 4C illustrates the Na⁺/K⁺-ATPase activity in *F. oxysporum* treated with various concentrations of nerol. The enzyme activity was markedly reduced in the nerol treatment groups in comparison to the control (*p* < 0.05). Specifically, the control group showed Na⁺/K⁺-ATPase activity at 168.46 U/mg prot, while the activities in the 0.01, 0.02, and 0.04 μL/mL nerol treatment groups were 80.26, 36.27, and 47.20 U/mg prot, respectively. These represent 47.64%, 21.53%, and 28.02% of the control’s activity.

#### 3.2.4. Disruption of Membrane Integrity by Nerol in *F. oxysporum*

**Figure 5:**
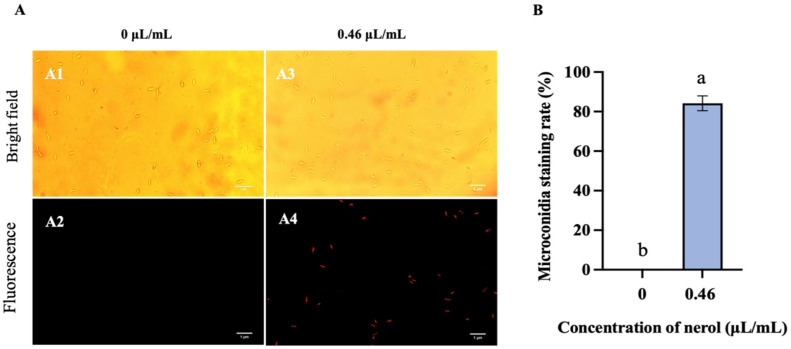
Nerol-induced disruption of *F. oxysporum* microconidia cell membranes visualized using fluorescence microscopy after PI staining. (**A**) indicate the fluorescence microscopy images results after PI staining. Fluorescence microscopy images showing PI-stained microconidia treated with 0 µL/mL (**A1**,**A2**) and 0.46 µL/mL nerol (**A3**,**A4**). The scale bar is 1 µm. PI staining rate of microconidia at different nerol concentrations (**B**). Histograms represent the mean (n = 3) ± standard error. Values accompanied by the different letters are significantly different (*p* ≤ 0.05) according to the Tukey test.

Microconidia treated with 0.46 μL/mL nerol exhibited red fluorescence, while control group microconidia showed low red fluorescence (Figure 5A). The microconidia staining rate in the treatment group reached 84.22% (Figure 5B), confirming membrane destruction by 0.46 μL/mL nerol treatment.

**Figure 6:**
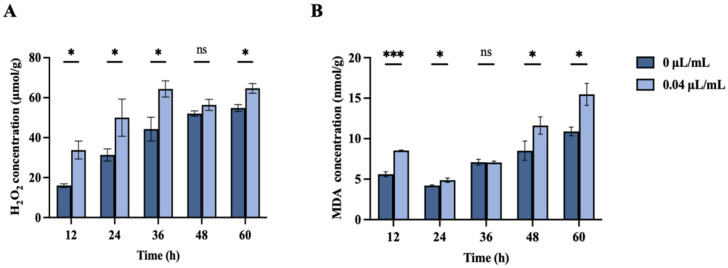
Effects of nerol at concentrations of 0 µL/mL and 0.04 µL/mL on H_2_O_2_ content (**A**) and MDA content (**B**) in *F. oxysporum* mycelia over different treatment times (0 µL/mL as control). Bars represent the mean ± standard error (n = 3). *, *** indicate statistically significant differences at *p* < 0.05 and *p* < 0.001, respectively. “ns” = no significant difference.

The H_2_O_2_ concentration in *F. oxysporum* treated with nerol for 12, 24, 36, and 60 h significantly increased in comparison to the control (Figure 6A, *p* < 0.05). After 36 h of treatment, the H_2_O_2_ content reached its peak value of 64.67 μmol/g.

The MDA concentration in *F. oxysporum* treated with nerol for 12, 48, and 60 h showed a significant increase relative to the control (Figure 6B, *p* < 0.05). MDA content gradually increased from 4.20 nmol/g to 10.89 nmol/g over 24 to 60 h in the control group, while in the treatment group, it rose from 4.88 nmol/g to 15.47 nmol/g during the same period. Treatment with 0.04 μL/mL nerol for 60 h resulted in the highest MDA content of 15.47 nmol/g, 1.42 times that of the control.

#### 3.2.5. Impact of Nerol on Antioxidant Enzyme Activity in *F. oxysporum*

**Figure 7:**
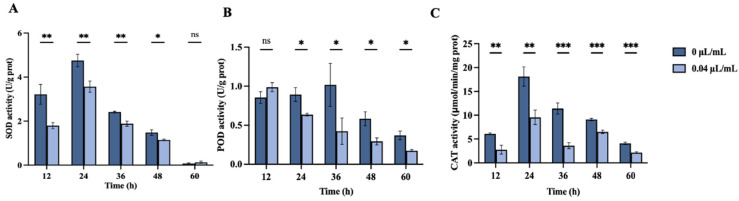
Effects of nerol at concentrations of 0 µL/mL and 0.04 µL/mL on the activities of SOD (**A**), POD (**B**), and CAT (**C**) in *F. oxysporum* mycelia over different treatment times (0 µL/mL as control). The bars represent the mean ± standard error (n = 3). *, **, *** indicate statistically significant differences at *p* < 0.05, *p* < 0.01, and *p* < 0.001, respectively. “ns” = no significant difference.

Figure 7 illustrates the impact of nerol at concentrations of 0 µL/mL and 0.04 µL/mL on SOD, POD, and CAT activities in *F. oxysporum* over time points of 12, 24, 36, 48, and 60 h.

SOD activity initially increased and subsequently decreased throughout the treatment period (Figure 7A). SOD activity was significantly reduced relative to the control (0 µL/mL) at 12, 24, 36, and 48 h (*p* < 0.05). At 12 h, SOD activity in the treatment group was 1.80 U/mg prot, representing 55.81% of the control’s activity (3.22 U/mg prot). At 60 h of treatment, SOD activities in both groups reached their lowest levels, with no significant difference between them (*p* > 0.05).

POD activity showed no significant difference between the treatment and control groups at 12 h (Figure 7B, *p* > 0.05). However, it significantly decreased in the treatment group at 24, 36, 48, and 60 h relative to the control (*p* < 0.05), with the highest inhibition of 58.36% observed at 36 h.

The CAT activity of the control group was always higher than that of the treatment group over the period (Figure 7C, *p* < 0.01). Specifically, at 36 h of treatment, CAT activity in the control was 11.41 μmol/min/mg prot, whereas, in the treatment group, it was only 3.60 μmol/min/mg prot, representing 31.58% of the control’s activity.

#### 3.2.6. Effect of Nerol on PG, PL, and EG Activities of *F. oxysporum*

**Figure 8:**
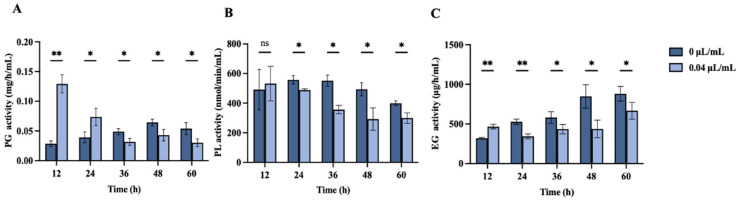
Effects of nerol at concentrations of 0 µL/mL and 0.04 µL/mL on PG activity (**A**), PL activity (**B**), and EG activity (**C**) in *F. oxysporum* mycelia over different treatment times (0 µL/mL as control). The bars represent the mean ± standard error (n = 3). *, ** indicate statistically significant differences at *p* < 0.05 and *p* < 0.01, respectively. “ns” = no significant difference.

Figure 8 illustrates the impact of nerol at concentrations of 0 µL/mL and 0.04 µL/mL on PG, PL, and EG activities in *F. oxysporum* over time points of 12, 24, 36, 48, and 60 h.

PG activity in the treatment group was markedly elevated relative to the control (0 µL/mL) at 12 and 24 h (Figure 8A, *p* < 0.05). However, PG activity in the treatment group at 36, 48, and 60 h was significantly reduced relative to the control (*p* < 0.05). Over time, PG activity in the treatment groups demonstrated a decreasing trend.

PL activity remained unaffected after 12 h of nerol exposure, with no significant difference from the control (Figure 8B, *p* > 0.05). However, at 24, 36, 48, and 60 h, PL activity in the treatment group was markedly lower compared to the control (*p* < 0.05).

At 12 h, EG activity increased significantly in the treatment group (*p* < 0.05). However, at later time points (24, 36, 48, and 60 h), EG activity in the treatment group was markedly suppressed compared to the control (Figure 8C, *p* < 0.05). At 48 h, EG activity in the treatment group was only 51.34% of that of the control group.

## 4. Discussion

Nerol, a linear acyclic monoterpene alcohol compound extracted from plants, has demonstrated significant potential in various fields such as medicine, food, and microbiology due to its efficacy and non-toxic nature [26,35,36,37]. Nevertheless, there has been limited exploration of the antifungal activity and mechanisms of nerol against fungal pathogens associated with forest diseases. In this study, nerol effectively inhibited the mycelial growth of *F. oxysporum*, *P. neglecta*, and *V. mali*, with *F. oxysporum* showing the highest sensitivity. This observation suggests that nerol possesses broad-spectrum antifungal activity and holds promise as a multifunctional natural fungicide. Additionally, nerol significantly inhibited the mycelial growth and spore germination of *F. oxysporum* on both PDA and PDB media in a concentration-dependent manner. These findings are consistent with the inhibitory efficacy of nerol observed on *Ceratocystis fimbriata* and *Aspergillus niger* in previous studies [26,28]. To elucidate the antifungal mechanism of nerol against *F. oxysporum*, we employed scanning electron microscopy to study morphological and structural changes in the mycelia. SEM images clearly demonstrated that nerol severely altered the morphology of *F. oxysporum* mycelia, causing twisting, swelling, flattening, and even breakage. The surface of the treated mycelia exhibited numerous distinct folds, and the mycelial boundaries appeared blurred. Such structural damage to fungal mycelia by plant-derived fungicides has been documented in numerous studies. For instance, Pan et al. [38] reported that matrine (a quinolizidine alkaloid isolated from the Chinese herb *Sophorae flavescentis*) induced the twisting and shrinking of *Botryosphaeria dothidea* mycelia, along with the release of intracellular contents, membrane wrinkling, and cytoplasmic contraction. Similarly, juniper essential oil was observed to cause the deformation of *Botrytis cinerea* mycelia. Several studies have suggested that surface roughness, the collapse of mycelia, and plasmolysis may be associated with the increased permeability of the mycelial cell membrane, resulting in the release of intracellular macromolecules [39,40]. Based on these observations, we hypothesize that the cell membrane represents one of the primary focuses of nerol’s antifungal action against *F. oxysporum*. Nerol disrupts the mycelial cell membrane, resulting in abnormal mycelial morphology. This hypothesis is further supported by our assessments of cell membrane damage.

The integrity of microbial plasma membranes is vital for preserving their typical morphology and fundamental life activities [41]. These functions include regulating the internal environment’s balance, ensuring selective permeability, and synthesizing key virulence-related compounds [42]. Many antifungal agents primarily target the cell membrane to exert their antifungal effects. For instance, tetramycin disrupts the plasma membrane integrity of *Alternaria alternata*, leading to the release of intracellular materials [43]. Similarly, natural antifungal agents such as tea tree oil [44], *o*-vanillin [45], and the mixed use of thymol and salicylic acid [46] all act by damaging fungal cell membranes. When the cell membrane structure is compromised, it loses its selective permeability, allowing the release of crucial intracellular components like nucleic acids, proteins, and ions into the extracellular matrix [47,48]. This disruption can suppress mycelial growth and ultimately cause cell death [49]. In our research, it was noted that nerol markedly increased the release of intracellular soluble proteins compared to the minimal leakage noted in the control group. This finding aligns with the results of conductivity tests and Na⁺/K⁺-ATPase activity assays. Normally, the ion homeostasis of cell membranes is maintained by ion-channel proteins and ATPases in the membrane [50]. The data we have obtained indicate that nerol affects the activity of Na⁺/K⁺-ATPase, leading to plasma membrane damage. This damage compromises the membrane’s selective permeability, resulting in a substantial release of soluble proteins and high-concentration ions into the extracellular matrix, which significantly increases extracellular conductivity and soluble protein levels. Previous studies have reported similar outcomes, where treatments with citral on *Penicillium italicum* [51], esculetin on *Phytophthora capsica* [52], and tea tree oil on *Botrytis cinerea* [53] all resulted in the release of intracellular proteins and ions due to impaired cell membrane permeability.

Based on several studies, the excessive buildup of reactive oxygen species (ROS), which induces lipid peroxidation, is recognized as an important cause of increased cell membrane permeability [54,55], with H_2_O_2_ and MDA levels serving as crucial indicators reflecting the extent of lipid peroxidation in cell membrane [56,57]. In our work, we observed a notable rise in H_2_O_2_ and MDA content in *F. oxysporum* following nerol treatment, indicating that nerol promotes the buildup of ROS within the fungal cells, exacerbating the extent of membrane lipid peroxidation and causing severe disruption to the plasma membrane. Research suggests that many plant-derived compounds achieve their fungicidal effects by inducing lipid peroxidation in fungi, such as *Thymus vulgaris* CT *carvacrol* and *Thymus vulgaris* CT *thymol* against *Rhizopus stolonifera* [58], nerol against *Aspergillus flavus* [59], and cinnamaldehyde against *Alternaria alternata* [60], among others. Conversely, the MDA level in *A. flavus* treated with cinnamaldehyde was reduced [61]. Furthermore, using a fluorescence microscope, we investigated the impact of nerol on the integrity of *F. oxysporum* microconidia cell membranes. Compared to the control group, microconidia exposed to nerol contained significantly more red fluorescence, indicating that a large amount of PI dyed the microconidia. Similar phenomena of substantial PI influx were observed in the study on *Aspergillus niger* treated by perillaldehyde [62]. Our findings suggest that nerol disrupts the integrity of *F. oxysporum* cell membranes, increases membrane permeability, leads to leakage of cellular components within hyphae, and affects fungal growth and development. This may explain the fungicidal efficacy of nerol against *F. oxysporum*. These results align with those reported by Li et al. [28], who demonstrated that nerol inhibits *Ceratocystis fimbriata* by disrupting its cell membrane.

Additionally, we assessed SOD, POD, and CAT activities in *F. oxysporum*. SOD, POD, and CAT are crucial elements of the antioxidant defense system in organisms, collectively playing a vital role in scavenging oxygen free radicals to prevent excessive buildup of ROS and mitigate the damage caused by lipid peroxidation [63]. It has been reported that the prolonged exposure of organisms to adverse conditions inhibits the activity of antioxidant enzymes in the body, resulting in the accumulation of ROS and oxidative stress and ultimately hindering the normal growth of fungi [64,65]. In our study, nerol likely inhibits antioxidant enzyme activities in *F. oxysporum* and diminishes the efficiency of oxygen radical scavenging, leading to the accumulation of H_2_O_2_, membrane lipid peroxidation, and the suppression of vital cellular activities. However, not all plant-derived compound treatments yield similar results in fungal antioxidant enzymes. For instance, treatment with 6-Methylcoumarin reduces CAT activity and increases SOD activity in *Valsa mali* [31], while 1-octanol treatment enhances CAT and SOD activities in *Aspergillus flavus* [66].

The plant cell wall is a vital barrier protecting plants from pathogenic fungi, with pectin and cellulose being crucial components of its structure [67]. During the process of fungal infection, pathogens disrupt the plant cell wall by producing various plant cell wall-degrading enzymes (PCWDEs), thereby compromising the plant’s defense mechanisms and accelerating tissue invasion. PCWDEs exemplified by polygalacturonase (PG), pectin lyase (PL), and endoglucanases (EG) are closely associated with the pathogenicity of fungi [68,69,70,71]. Like most plant pathogenic fungi, *F. oxysporum* produces multiple PCWDEs during host invasion to breach the plant’s defense barriers [72,73]. Nerol may reduce the activity of PCWDEs in *F. oxysporum*, thereby weakening its ability to degrade the host cell wall and diminishing its pathogenicity. This finding aligns with the findings presented by Li et al. [4] in their research on the antifungal effects of phytic acid against *F. oxysporum*, where it was observed that PA reduces pectinase and cellulase activities in *F. oxysporum*.

This study demonstrates the in vitro antifungal activity of nerol against *F. oxysporum*, *P. neglecta*, and *V. mali*. Future research will focus on exploring its potential in practical applications by conducting in vivo experiments with seedlings to further validate its efficacy and safety. This will support the use of nerol as an eco-friendly fungicide in agriculture and forestry. To address nerol’s limitations in water solubility and environmental stability, future studies may investigate advanced formulation techniques such as nanoparticle encapsulation, particularly in combination with other natural compounds. The use of suitable additives, such as 1% Tween-80, has been shown to enhance the solubility of nerol while maintaining plant safety. Additionally, evaluating the scalability and cost-effectiveness of nerol production is essential to determine its feasibility for large-scale applications. Overall, despite existing challenges, nerol has the potential to become a significant alternative to chemical fungicides, warranting further research and development.

## 5. Conclusions

In conclusion, nerol exhibited significant inhibitory effects on the mycelial growth of *F.oxysporum*, *P. neglecta*, and *V. mali*, with the strongest inhibition observed against *F. oxysporum*. Nerol caused severe disruptions in the normal morphology of the fungal hyphae. Additionally, nerol increased the permeability of the *F. oxysporum* cell membrane and compromised its integrity, leading to the leakage of intracellular components. Nerol also inhibited the activities of antioxidant enzymes SOD, POD, and CAT, inducing oxidative damage to the cell membrane. Moreover, nerol significantly suppressed the activities of PG, PL, and EG, thereby reducing the pathogenicity of *F. oxysporum*. These findings suggest that nerol, a novel plant-derived natural compound, has the potential to replace traditional chemical fungicides as an eco-friendly antifungal agent.

## Data Availability

Data are contained within the article.
